# Evaluation of the Safety of a Plant-Based Infant Formula Containing Almonds and Buckwheat in a Neonatal Piglet Model

**DOI:** 10.3390/nu14071499

**Published:** 2022-04-02

**Authors:** Fernanda Rosa, Brooke Yelvington, Nathan Terry, Patricia Tripp, Hoy E. Pittman, Bobby L. Fay, Taylor J. Ross, James D. Sikes, Jessica B. Flowers, Fabiana Bar-Yoseph, Laxmi Yeruva

**Affiliations:** 1 United States Department of Agriculture-Agriculture Resaarch Service, Arkansas Children’s Nutrition Center, Little Rock, AR 72202, USA; ftrindad@ttu.edu (F.R.); yelvingtonbe@archildrens.org (B.Y.); terrynh@archildrens.org (N.T.); tripptm@archildrens.org (P.T.); pittmanhe@archildrens.org (H.E.P.III); faybl@archildrens.org (B.L.F.); rosst@archildrens.org (T.J.R.); sikesjd@archildrens.org (J.D.S.); 2 School of Veterinary Medicine, Texas Tech University, Amarillo, TX 79415, USA; 3 Technical Services Envigo, Teklad Diets, Madison, WI 53713, USA; jessicaflowers.phd@gmail.com; 4 Else Nutrition GH, Tel Aviv 6971070, Israel; fabianab@elsenutrition.com

**Keywords:** infant formula, plant-based, piglet model, metabolism

## Abstract

A randomized neonatal piglet trial was conducted to evaluate the safety and the effects of a plant-based formula containing almonds and buckwheat as the main ingredients on growth and plasma parameters. From postnatal day (PND) 2 to 21, the piglets were fed a dairy-based milk formula (Similac Advance) or a plant-based formula (Else Nutrition) and all piglets were euthanized at day 21. No diarrhea was observed after PND 8 and all the piglets completed the trial. Body growth, kcal intake, the complete plasma count parameters and hematological parameters were within the reference range in both groups. Organ growth and development was similar between the two groups. Plasma glucose was higher in the dairy-based-fed piglets relative to the plant-based at 2 weeks of age. Liver function biomarkers levels were greater in the plasma of the plant-based compared to the dairy-based fed group. In addition, calcium levels were higher in the plant-based fed piglets at 1 week of age. Thus, the plant-based formula tested in this study was well tolerated by the piglets and supported similar growth compared to dairy-based milk formula. Therefore, the results support the safety of the tested plant-based infant formula during the neonatal period in comparison to the dairy-based formula fed group.

## 1. Introduction

Human milk is the gold standard for the neonatal feeding, growth, and health of infants worldwide [1,2]. Infant formulas are a safe nutritional alternative to support growth and normal development in infants, whenever human milk feeding is not possible [3]. Most infant formulas are produced with bovine milk as the main source of protein and carbohydrates. However, different approaches to the infant formula composition have been evaluated to address gastrointestinal lactose intolerance and food allergies to the standard cow-protein-based formulas. Such approaches include the hydrolyzed-protein formulas, lactose-free or lactose reduced formulas, and soy-protein-based formulas [4,5]. In the USA, approximately 12% of the infant formula market are soy-based formulas [6]. Different studies have reported that soy-based formulas support the normal growth and development of full-term infants [7,8,9,10]. Recently, a randomized clinical trial reported that soy-based formulas can reduce gastrointestinal symptoms in infants with cow’s milk intolerance, including the reduction in gas, crying, and a higher formed stool in healthy infants [11]. Although soy-protein formulas are recommended as a nutritional alternative to human milk in full-term infants [12], some concerns on the risk of phytoestrogen concentration in soy-based formulas have been raised [13,14], as well as the lower bone mineralization observed in infants fed a soy-based formula during the first year of life [15]. There is an increasing interest in plant-based alternatives due to allergies, intolerance, or for perceived health benefits or cultural values, which are leading some families to feed their babies plant-based “milks” that are not designed specifically for infant or young children and are deficient in nutrients [16,17]. Although certain nuts can be allergenic for some individuals and their use should be evaluated with caution for allergic individuals [18], the common protein sources in infant formulas, cow milk and soy, are major allergens. Moreover, recent guidelines recommend the early introduction of allergens to infants below the age of 12 months [19,20]. However, a nutritious plant-based formula with a different source of protein rather than soy-protein is needed due to the allergies to dairy milk protein, intolerance to lactose, and to provide an option to those following veganism practices.

The evaluation of the safety of infant formulas is essential to determine the impact of different ingredients on the infants’ health, nutritional status, and to prevent long-term metabolic disorders. In the present study, we compare the growth, organ development, and metabolic parameters in the plasma of piglets fed a dairy-based formula or a plant-based formula from postnatal day (PND) 2 until PND 21 in a controlled environment. We hypothesized that the plant-based formula that contains almond butter and buckwheat flour tested in this study provides an adequate nutritional source relative to the other infant formula alternatives in the market.

## 2. Materials and Methods

### 2.1. Experimental Design and Animal Care

The Institutional Animal Care and Use Committee (IACUC) at the University of Arkansas for Medical Sciences approved all animal care and experimental procedures (UAMS Institutional Animal Care and committee protocol# 4022). Piglets were purchased from a biomedical research farm (Oak Hill Genetics, Ewing, IL, USA), which meets all the requirements of Class “A” licensee by the United States Department of Agriculture (USDA). After birth, piglets were allowed to suckle at their respective sows for 48 h prior to being transported to the animal facility of the Arkansas Children’s Hospital Research Institute. Upon arrival, at postnatal day 2 (PND 2), 18 crossbred (Yorkshire/Landrace cross with some Duroc on the boar side) intact male and female piglets were randomly assigned into 2 dietary groups of approximately equal mean weight (*n* = 9/diet group): dairy-based infant formula (Similac Advance powder; Abbott Nutrition Abbott Laboratories, Columbus, OH, USA) or plant-based formula (Else Nutrition GH Ltd., Tel Aviv 6971070, Israel). At PND 2, piglets were trained to drink from nipples on a fixed schedule to provide 1.047 MJ/kg/day until sacrifice on day 21. Formula-fed piglets were individually housed, which allowed the monitoring of milk intake at each feeding and the evaluation of stool consistencies. This animal trial was completed in 3 replicates (6 piglets/replicate), with 3 piglets/diet selected from 2 different sows. All piglets were housed in the animal facility from PND 2 until PND 21. Piglets were observed daily during the course of the experimental study for any signs of stress by trained and experienced technical staff or by the investigators. No medication treatment was used during the trial. Two piglets (1/diet group) had diarrhea during the postnatal days 3–8. These piglets were treated with Pedialyte as per veterinarian recommendation and IACUC requirement to help with electrolyte loss. All the piglets completed the study.

### 2.2. Diet Groups

The plant-based formula was formulated and supplied by the Else Nutrition GH Ltd. (main ingredients: almond butter and buckwheat flour), while the dairy-based formula (Similac Advance powder) was purchased from the commercial company (Abbott Nutrition Abbott Laboratories, Columbus, OH, USA). To meet the nutritional requirements of growing pigs by the National Research Council (NRC) [21], both diets were supplemented with a blend of nutrients, which was formulated and produced by Envigo Teklad Diets (Madison, WI, USA). The complete list of nutrients in each formula is shown in Table 1. The formula powder was reconstituted fresh daily with water, safflower, and coconut oils to meet the daily desired caloric intake (based on the body weight) of 300 Kcals at first week, 200 Kcals at second week, and 175 Kcals at third week divided by the number of feeds. The feeding schedule was as follows: first week was every 2 h, second week was every 4 h, and third week was every 6 h.

### 2.3. Sample Collection and Measurements

Body weights were measured daily throughout the trial. Firstly, 2 h after morning feeding (e.g., 6 a.m.), blood samples were collected weekly starting at day 5 until 21 days of age, with piglets under anesthesia using a tight-fitting mask with a dose of 3–5% isoflurane and with oxygen between 0.8 to 1.5 L/min. Prior to blood collection, the neck was shaved and cleaned with 70% alcohol scrubs. The weekly blood draws were performed based on the circulating blood volume (CBV) of 7.5% and the estimated CBV of 60 mL/kg. At day 21, blood collection was a terminal bleed. Blood was collected via cardiac puncture with an 18-gauge 3-inch needle and 20 mL syringes, while piglets were under anesthesia. On day 21 after exsanguination, several organs and tissues were collected for further analysis, including gastrointestinal tract tissues (duodenum, jejunum, ileum, proximal and distal colon), gastrointestinal tract contents, as well as mesenteric lymph nodes, spleen, liver, and kidney.

### 2.4. Metabolic and Trace Mineral Profiling in Plasma

At the weekly blood draws, approximately 2.5 mL of whole blood was collected into K_2_EDTA vacutainer tubes (BD Biosciences, Cat#367844), 2.0 mL into lithium heparin vacutainer tubes (BD Biosciences, Cat#367880, Franklin Lakes, NJ, USA), and 2.5 mL into blood RNA tubes (BD, PAXgene^®^, Cat#762165, Franklin Lakes, NJ, USA). In addition, using 2 drops of whole blood per piglet, 2 slide smears were collected. Plasma samples were obtained by spinning the lithium heparin tubes at 1500× *g* for 10 min at 4 °C. A total of 500 uL of plasma, K_2_EDTA tubes, and the blood smear samples were submitted to the Veterinary Medical Diagnostic Laboratory at Texas A&M University (College Station, TX, USA) for metabolic profiling, mineral panel, and complete blood count (CBC), respectively. Additionally, plasma samples were submitted to the Eurofins Craft Technologies Laboratory (Wilson, NC, USA) for plasma insulin measurements using a quantitative porcine insulin ELISA assay. At day 21, the blood (~45 mL) was collected into serum vacutainer tubes (BD Biosciences, Cat#368045, Franklin Lakes, NJ, USA) as well as into blood RNA tubes for further analysis. Serum tubes were incubated at room temperature for approximately 20 min and then centrifuged at 1500× *g* for 10 min at 4 °C. Serum aliquots were stored at −80 °C until further processing.

### 2.5. Statistical Analysis

This study was designed as a 2 × 2 factorial arrangement of treatments (dairy-based and plant-based). Some study responses were measured repeatedly over time, while some comparisons were performed at the endpoint time. Therefore, the proposed study design with 9 animals per group with roughly equal gender representation and equal mean body weight for the piglets at PND 2 (enrollment day) has approximately 80% power to detect 1.25 standard deviation units between the diet groups testing at the 5% level of significance. The statistical analysis was conducted in the SAS version 9.4 (Cary, NC, USA) and GraphPad Prism software version 9.1.2 (San Diego, CA, USA). Group comparisons of body weights, calories intake, and plasma parameters overtime and their interactions (Group × Time) were analyzed by repeated measures analysis of variance (ANOVA) followed by Sidak’s multiple-comparisons adjusted *p*-value. Group comparisons for the organ weights and length were assessed by two-tailed Mann–Whitney test (non-parametric test). Statistical significance was declared at *p* ˂ 0.05.

## 3. Results

### 3.1. Body Weights

**Figure 1:**
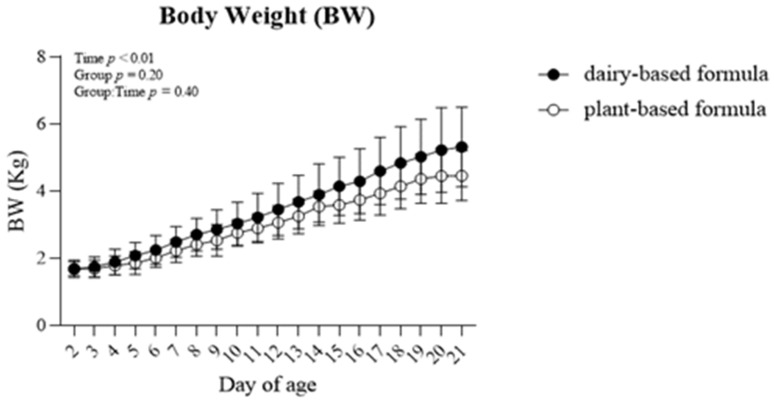
Average body weight (BW; Kg) per day of piglets fed either a dairy-based (Similac Advance powder) or a plant-based (Else nutrition powder) formula (*n* = 9/group) from postnatal day (PND) 2 until PND 21.

No group × time interaction (*p* = 0.40) was observed for body weight (BW) within the repeated-measures analysis (Figure 1). However, the BW increased in both dietary groups overtime (*p* < 0.01).

### 3.2. Caloric Intake

**Figure 2:**
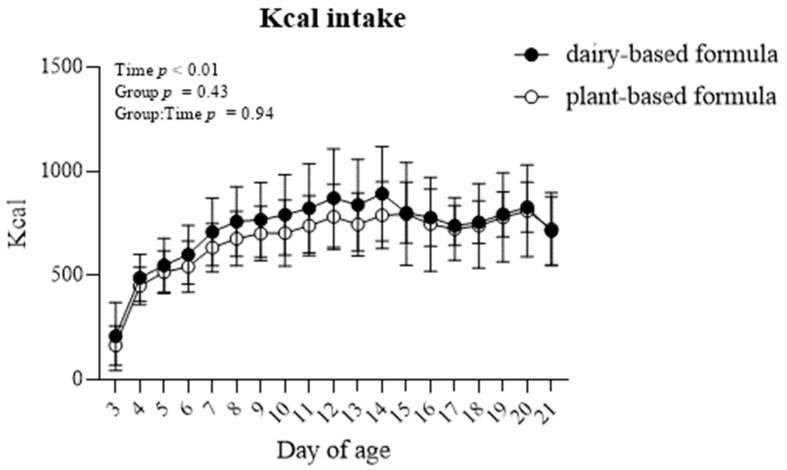
Average formula intake (Kcal) per day of piglets fed either a dairy-based (Similac Advance powder) or a plant-based (Else nutrition powder) formula (*n* = 9/group) from postnatal day (PND) 2 until PND 21.

The formula caloric intake (Kcal) increased overtime in both groups of piglets (*p* < 0.01). Overall, the Kcal intake was similar between the dairy-based and plant-based formula-fed piglets (Figure 2).

### 3.3. Organ Development

**Figure 3:**
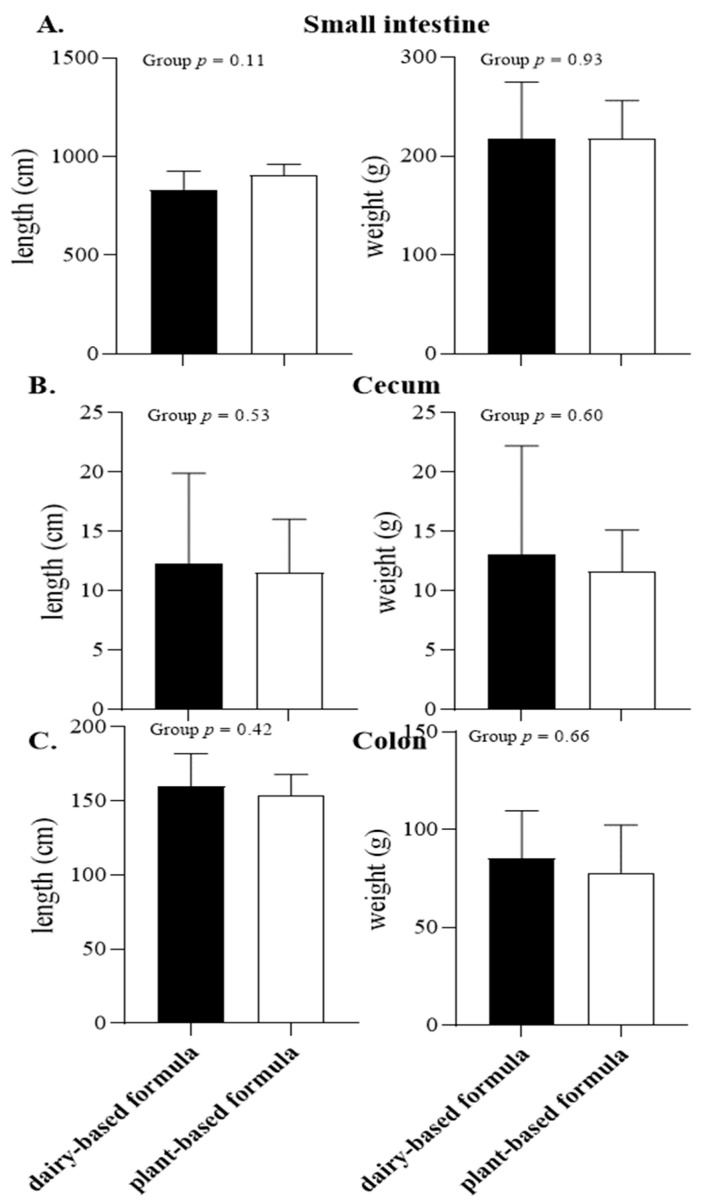
Absolute length (cm) and organ weight (g) of small intestine (**A**), cecum (**B**), and colon (**C**) of 21-day-old piglets fed either a dairy-based (Similac Advance powder) or a plant-based (Else nutrition powder) formula (*n* = 9/group) from postnatal day (PND) 2 until PND 21.

The absolute intestinal length and weight are shown in Figure 3. No difference between the diet groups was observed for the small intestine, cecum, and colon length and weights (Figure 3A–C, respectively). In addition, the absolute organ weights for spleen, pancreas, kidneys, liver, adrenals, and the reproductive tract did not differ between the diet groups at PND 21 (Table 2).

### 3.4. Plasma Biomarkers

#### 3.4.1. Metabolic Profiling

**Figure 4:**
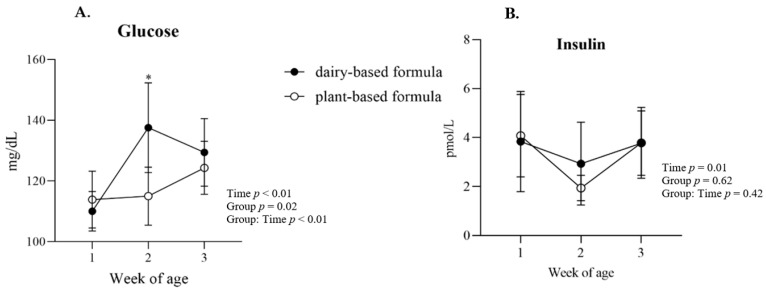
(**A**) Plasma glucose and (**B**) insulin (average per group) at 1, 2, and 3 weeks of age of piglets fed either a dairy-based (Similac Advance powder) or a plant-based (Else nutrition powder) formula (*n* = 9/group) from postnatal day (PND) 2 until PND 21. * Represents the statistical difference between diet groups at the given time point.

**Figure 5:**
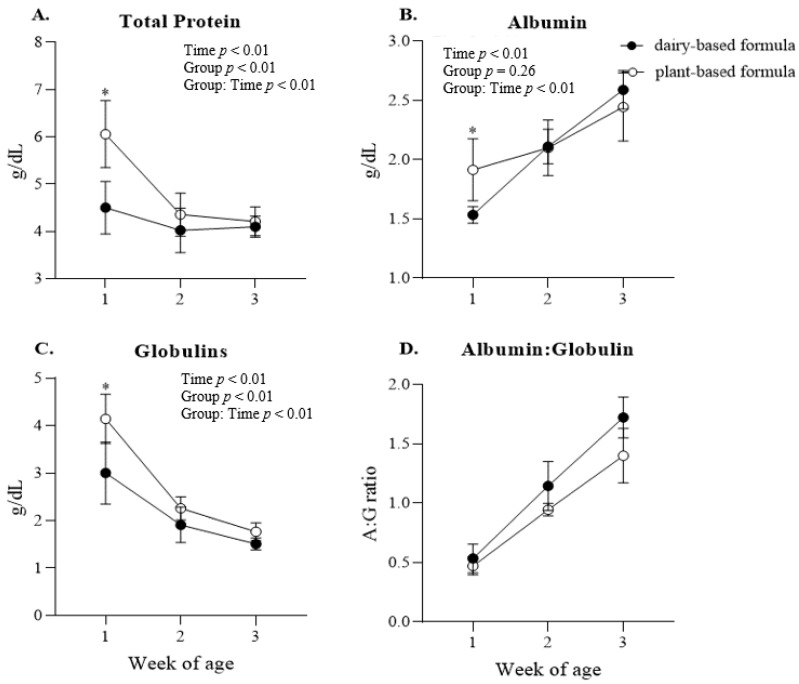
Average serum (**A**) total protein, (**B**) albumin, (**C**) globulins, (**D**) albumin:globulin ratio at 1, 2, and 3 weeks of age of piglets fed either a dairy-based (Similac Advance powder) or a plant-based (Else nutrition powder) formula (*n* = 9/group) from postnatal day (PND) 2 until PND 21. * Represents the statistical difference between diet groups at the given time point.

Glucose: a group × time interaction (*p* < 0.01) was observed for plasma glucose, and the post hoc analysis revealed a higher glucose concentration in the plasma of the dairy-based group relative to the plant-based group at 2 weeks of age (Figure 4A). Overall, plasma glucose was lower in the plant-based group compared to the dairy-based formula group overtime (117.8 vs. 125.7 mg/dL, group average, respectively). Insulin: No significant interaction or group effect was observed for the concentration of insulin in plasma. However, a time effect (*p* = 0.01) was identified for insulin levels (Figure 4B). Protein: a group × time interaction was observed for plasma total protein, albumin, and globulins (Figure 5A–C, respectively). This significant interaction was reflected in the greater levels of total protein, albumin, and globulins in the serum of plant-based formula-fed piglets relative to the dairy-based group at 1 week of age (*p* < 0.01). The albumin:globulin ratio (A:G) did not reach significance (*p* = 0.10).

**Figure 6:**
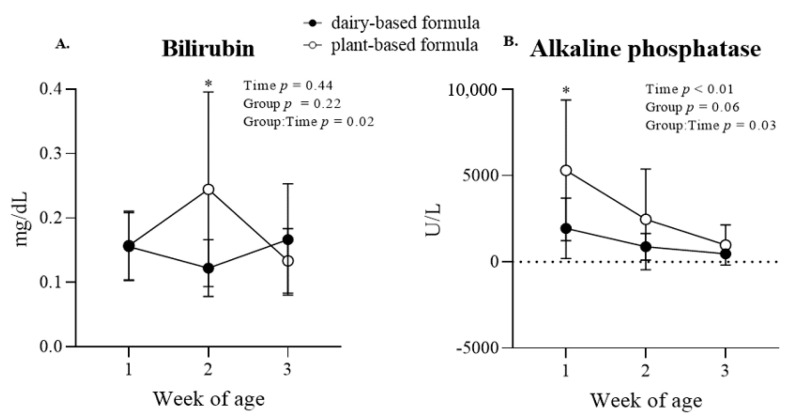
Plasma concentration of bilirubin (**A**) and alkaline phosphatase (**B**) at 1, 2, and 3 weeks of age of piglets fed either a dairy-based (Similac Advance powder) or a plant-based (Else nutrition powder) formula (*n* = 9/group) from postnatal day (PND) 2 until PND 21. * Represents the statistical difference between diet groups at the given time point.

Liver function biomarkers and enzymes: a group × time interaction (*p* = 0.02) was observed for serum bilirubin, which was reflected in a higher bilirubin concentration (*p* < 0.01) in the plasma of the plant-based-fed piglets compared to the dairy-based group at 2 weeks of age (Figure 6A). Similarly, alkaline phosphatase levels decreased over time (*p* < 0.01), and a group × time interaction (*p* = 0.03) was observed, revealing higher levels (*p* = 0.01) in the plasma of the piglets fed a plant-based formula relative to the dairy-based group at first week of age (Figure 6B). No group × time interaction or group effect was observed for the enzymes creatinine kinase (CK), aspartate aminotransferase (AST), and alanine transferase (ALT) (Table 3). However, a time effect (*p* < 0.01) was observed for CK, which increased overtime in both groups. No other effects were detected for these enzymes (Table 3).

Electrolytes and kidney function: For both the sodium and chloride electrolytes, time and group effects were detected (Table 4). A group × time interaction (*p* < 0.01) was observed for the concentration of sodium, which was reflected in higher levels at the first week of age in the plasma of dairy-based-fed piglets relative to the plant-based diet group (*p* < 0.01; Table 4). No group × time interaction was observed for the concentration of chloride (Table 4). No main effects were observed for the plasma levels of potassium and for the sodium:potassium ratio in any of the diet groups (Table 4). A time effect was observed for the kidney function biomarker creatinine (*p* < 0.01), which increased from w2 to w3 in the plasma of all piglets regardless of the diet (Table 4). In contrast, no effects were observed for the plasma urea nitrogen concentration (BUN).

#### 3.4.2. Mineral Panel

The average concentration of minerals in the plasma is shown in Table 5. A group × time interaction was observed for the concentration of calcium, which was reflected in the higher levels of calcium in the plant-based-fed piglets compared to the dairy-based-fed group at the first week of age (*p* = 0.02). The levels of phosphorus and copper increased (*p* < 0.01) overtime in the plasma of all piglets regardless of the diet (Table 5). In contrast, the concentration of zinc decreased with time (*p* < 0.01) in the plasma of all piglets (Table 5).

#### 3.4.3. Complete Blood Count

**Figure 7:**
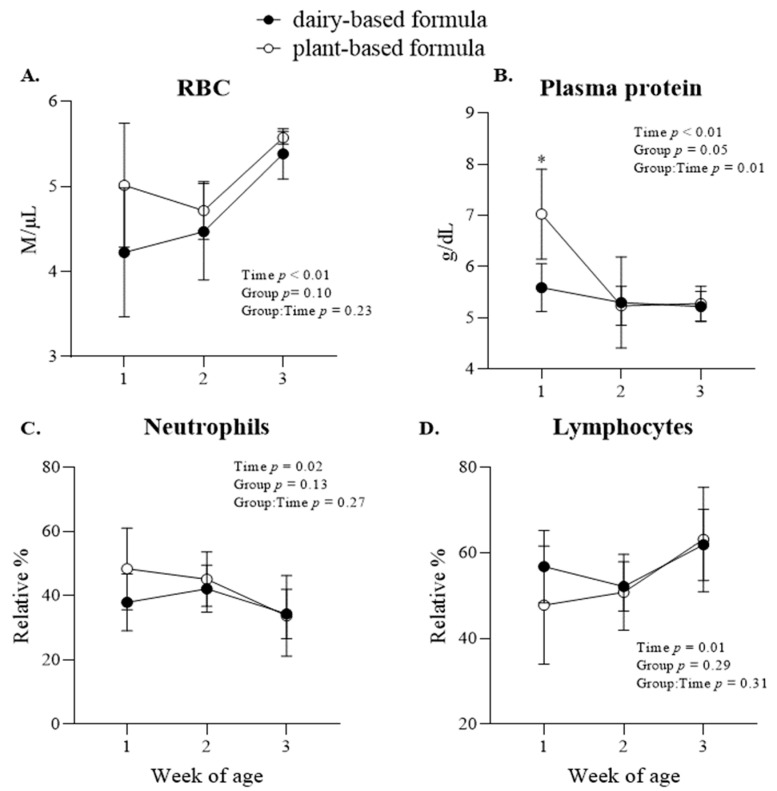
Plasma cell count variables: red blood cell—RBC (**A**), plasma protein (**B**), relative (%) neutrophils (**C**) and lymphocytes (**D**) at 1, 2, and 3 weeks of age of piglets fed either a dairy-based (Similac Advance powder) or a plant-based (Else nutrition powder) formula (*n* = 9/group) from postnatal day (PND) 2 until PND 21. * Represents the statistical difference between diet groups at the given time point.

The concentration of red blood cells increased in all piglets from 2 to 3 weeks of age (Figure 7A). A group × time interaction (*p* = 0.01) was observed for the plasma protein, which had higher levels in the plasma of the plant-based-fed piglets compared to the dairy-based-fed piglets at 1 week of age (Figure 7B). Overall, the relative % of neutrophils in plasma decreased, while the relative % of lymphocytes increased from the first week of age to 3 weeks of age in all piglets (Figure 7C,D). Group × time interaction effects were also observed for the concentration of hemoglobin and % hematocrit, which were reflected in higher levels in the plant-based group relative to the dairy-based group at 1 week of age (Table 6). Additionally, the absolute lymphocytes (k/µL) increased (*p* = 0.02) as the piglets grew regardless of neonatal diet (Table 6).

## 4. Discussion

Human milk is the best nutrition for infants, but many infants are fed infant formula due to maternal choice or other reasons. The increasing interest in plant-based feeding options for infants and young children led to the development of alternatives whose safety should be verified.

The present study demonstrated that the consumption of the plant-based formula was well tolerated (no fever observations, similar fecal consistencies, and no signs of prolonged diarrhea events) by piglets during the first 3 weeks of life. Furthermore, the similar observations in body weight and kcal intake throughout the experiment between the dairy-based and plant-based formula fed animals showed that the plant-based formula utilized in the current study also supported adequate growth in those piglets. The daily body weight measured in all the piglets was in line with the growth reported in sow-fed piglets during the neonatal phase [22,23,24] or soy-fed piglets reported by others [25], and in line with our previous findings, in which piglets received pasteurized human milk, dairy-based milk formula or sow-fed piglets [26]. Additionally, the gastrointestinal tract development as well as that of the organs examined were similar between both dietary groups and are comparable with neonatal piglets that received a dairy-based milk formula [27] or soy formula [28] for 21 days. Moreover, all plasma measures during the study are within the normal range reported for this age group, including CBC [22], chemistry [29], minerals [30] and all liver enzymes.

Neonates have high variability in several plasma biomarkers due to the accelerated growth and physiological adaptations during this period [31]. Therefore, there is a different reference range for plasma metabolites of neonates, young animals, and adults. For instance, bilirubin, which is the end product of the red blood cells catabolism, has been reported to be higher than the rate in adults due to the increased red blood cell turnover in newborns [32]. Serum alkaline phosphatase (ALP) is also 2–3 times higher in the plasma of neonates compared to healthy adult subjects [33], and its activity is commonly used as a clinical biomarker of bone metabolism and liver function; such processes include the transport of phosphates and calcium into the intestinal tissues, and bone mineralization [34].

The hematological variables measured in the present study are in accordance with the reference intervals for sow-fed piglets at a similar age (5 and 30 days old) and housed in a controlled environment [22]. Our CBC analysis showed a decrease in neutrophil percentage with age, while an increase in the percentage of lymphocytes was observed from 1 to 3 weeks of age in all piglets. These findings are consistent with the CBC reported in term infants in whom the neutrophil count peaked at the first 24 h of life and then decreased by 3 days of age [35,36]. Taken together, our data do not suggest an adverse effect of feeding a plant-based formula to neonatal piglets on the circulating cells. As calcium absorption and bone mineralization is of high importance in young infants and clinical trials have reported a lower bone mineralization in infants fed a soy-protein-based formula at 3 months of age [15], the difference in calcium observed in the first week may worth further examination of calcium metabolism, or bone mineralization.

Circulating proteins that are produced by the hepatocytes serve as liver function biomarkers [37]. Our trial demonstrated that plant-based-fed piglets had statistically higher albumin, total protein, and globulins concentration at 1 week of age compared to the dairy-based group. These levels reported in the plasma of the plant-based-fed piglets are higher than the levels reported by other studies that fed swine milk formula supplemented with 3′-siallylactose sodium salt to piglets from 2 to 21 days of age [38]. However, as the parameters are within normal range, probably there is no clinical significance to these observations.

The normal concentration of glucose in the plasma of newborn infants is 2.5 mmol/L (45 mg/dL) to 7.0 mmol/L (126 mg/dL). Though the average measured glucose levels in this study are within this range, more piglets in the dairy-based group had plasma glucose levels higher that the above as compared to the plant-based group. In addition, no difference in insulin levels were noted between the two diet groups. The differences observed may point to a more stable glucose metabolism in the plant-based group, a direction worth of further exploration.

One concern of plant-based products is around the antinutrients and especially phytate content decreasing the bioavailability of minerals and trace elements. Thus, the results reveal that the nutrient absorption was not compromised in the plant-based group, as shown by the similarity in caloric intake, growth pattern, and mineral plasma concentrations, which is of high significance to demonstrate the non-inferiority of this formula.

Pigs as an animal model for biomedical research have been extensively used, among other reasons, due to the physiological and anatomical similarities between pigs and humans [39,40]. Additionally, piglets during the first 3 weeks of life (i.e., neonatal phase) have been used to translate observations to human neonates due to the similar physiology and microbiota establishment and digestibility upon neonatal diet feeding between pigs and infants [41,42,43]. The safety of many infant formula ingredients was demonstrated in this model. Thus, this study used a neonatal piglet model to examine the safety and efficacy of a plant-based formula with almonds and buckwheat as the main ingredients, but the neonatal porcine model used in this study has some limitations. Piglets were enrolled in the trial at 2 days of age by which time the colostrum quality and amount consumed are unknown and they were from different sows. We acknowledge that such limitations might have introduced some baseline variation that might affect the findings. Additionally, to meet the energy and nutrient requirements for growing pigs, both diets were calculated to meet equal levels of additional protein and other compounds. This neonatal piglet experiment is a standard model to test neonatal diet and this porcine model has been used by different researchers [28,43,44,45], including for the evaluation of soy-protein-based formulas [46,47]. Despite the addition of nutrients to both formula diets, the infant formulas represent a significant component of the diets, and the fact that animals from both diets were maintained in a controlled environment (housed at the vivarium on a 12 h light/dark cycle), had similar growth, and both groups had similar kcal intake per day validate our results based on the neonatal diet offered to the piglets.

## 5. Conclusions

In summary, our study provides evidence that feeding a plant-based formula containing almond butter and buckwheat flour as the main ingredients is safe, well tolerated without significant adverse events, and supports the similar growth and development of piglets fed a dairy-based milk formula. Furthermore, any hematological parameters measured were within the reference ranges, further suggesting the safety of this the plant-based formula in a neonatal piglet model.

## Figures and Tables

**Table 1:** List of calculated nutrients for the dairy-based (Similac Advance powder) and plant-based formula (Else Nutrition powder) fed to piglets ^1^ (*n* = 9/group) from postnatal day (PND) 2 until PND 21. Additional information on the diet’s formulation can be found in Supplemental Table S1.

**Macronutrient**	**Dairy-Based Formula**	**Plant-Based Formula**
Protein, g/Kg	240.59	240.40
Carbohydrate, g/Kg	279.19	308.36
Fat, g/Kg	311.85	312.91
**Essential amino acids**	**Dairy-based formula**	**Plant-based formula**
Arginine, g/Kg	9.27	11.96
Cystine, g/Kg	8.22	6.29
Histidine, g/Kg	6.84	6.84
Isoleucine, g/Kg	14.56	12.92
Leucine, g/Kg	32.16	28.37
Lysine, g/Kg	25.41	20.68
Methionine, g/Kg	5.82	4.64
Phenylalanine, g/Kg	11.19	15.29
Threonine, g/Kg	12.81	10.79
Tryptophan, g/Kg	5.30	4.64
Valine, g/Kg	13.69	11.42
**Minerals ^2^**	**Dairy-based formula**	**Plant-based formula**
Ca, g/Kg	10.10	10.16
Cl, g/Kg	6.83	5.58
K, g/Kg	8.15	8.82
Mg, g/Kg	0.66	0.90
Na, g/Kg	5.06	5.11
P, g/Kg	8.68	8.68
Cr, mg/Kg	1.00	1.00
Cu, mg/Kg	8.26	9.17
Fe, mg/Kg	124.13	123.65
I, mg/Kg	0.35	0.98
Mn, mg/Kg	10.67	13.54
Mo, mg/Kg	0.15	0.15
Se, mg/Kg	0.38	0.38
Zn, mg/Kg	179.39	174.29
**Vitamins**	**Dairy-based formula**	**Plant-based formula**
B12, mg/Kg	0.03	0.05
B6, mg/Kg	7.25	11.73
Biotin, mg/Kg	0.31	0.50
Folic Acid, mg/Kg	2.38	3.02
Niacin, mg/Kg	55.99	62.28
Pantothenate, mg/Kg	25.77	54.26
Riboflavin, mg/Kg	9.78	17.58
Thiamin, mg/Kg	7.23	13.02
Vit A, IU/Kg	7101.48	11,160.00
Vit D, IU/Kg	1775.37	3096.00
Vit E, IU/Kg	36.02	154.20
Vit K, mg/Kg	0.94	1.01
**Other compounds**	**Dairy-based formula**	**Plant-based formula**
Linoleic acid, g/Kg	103.27	100.99
Choline, mg/Kg	1221.33	1221.30
Inositol, mg/Kg	114.76	750.00

^1^ A total of 204.5 g dairy-based and 199.6 g plant-based complete diet were used per L of prepared formula to give 1 Kcal/mL. Both formulas were mixed with safflower and coconut oils to make the complete diet. The complete diet had a caloric density of 4.89 and 5.01 Kcal/g for the dairy-based and plant-based formulas, respectively. ^2^ Ca = Calcium; P = Phosphorus; Na = Sodium; Cl = Chloride; Mg = Magnesium; K = Potassium; I = Iodine; Fe = Iron; Mn = Manganese; Mo = Molybdenum; Se = Selenium; Zn = Zinc; Cr = Chromium; Cu = Copper. Dairy-based formula—Similac Advance; Plant-based formula—Else Nutrition formula.

**Table 2:** Organ weights (g) of neonatal piglets at 21 days of age fed either a dairy-based or a plant-based formula. Data are presented as mean ± standard deviation (SD).

Organ ^1^	Dairy-Based Formula ^2^	Plant-Based Formula ^3^	*p*-Value ^4^
Spleen	8.47 ± 1.99	7.52 ± 1.95	0.37
Pancreas	7.51 ± 2.41	7.50 ± 2.78	0.71
Kidneys	33.38 ± 9.48	25.16 ± 2.30	0.10
Liver	142 ± 33	132 ± 25	0.55
Adrenals	0.76 ± 0.17	0.82 ± 0.32	0.74
Prostate	0.23 ± 0.05	0.15 ± 0.05	0.12
Left testicle	3.4 ± 0.82	3.52 ± 1.29	0.87
Right testicle	3.38 ± 0.72	3.45 ± 1.67	0.44
Uterine horn	3.26 ± 1.21	3.17 ± 0.55	0.79
Ovaries	0.12 ± 0.04	0.15 ± 0.07	0.99

^1^ Spleen, pancreas, kidneys, liver, adrenals (*n* = 9/group); prostate, left and right testicles (*n* = 4 dairy-based formula; *n* = 6 plant-based formula); uterine horn and ovaries (*n* = 5 dairy-based formula; *n* = 3 plant-based formula); ^2^ Similac Advance powder; ^3^ Else Nutrition powder; ^4^ *p*-value from the Mann–Whitney test.

**Table 3:** Group average per week of age (w1, w2, w3) of enzymes measured in the plasma of piglets fed either a dairy-based or a plant-based formula (*n* = 9/group) from postnatal day (PND) 2 until PND 21.

								*p* ^4^			*p*-Adjusted ^5^		
	Dairy-Based Formula ^1^			Plant-Based Formula ^2^			SEM ^3^	Time	Group	Group × Time	w1	w2	w3
Enzyme	w1	w2	w3	w1	w2	w3							
CK (U/L)	244.4	476.2	941.7	183.0	422.2	448.2	195.80	<0.01	0.11	0.14	0.99	0.99	0.03
AST (U/L)	30.78	36.89	43.67	38.77	47.22	29.33	9.87	0.47	0.83	0.10	0.81	0.61	0.34
ALT (U/L)	18.33	19.33	22.67	21.73	24.67	20.78	2.80	0.55	0.17	0.15	0.55	0.14	0.86

^1^ Similac Advance powder; ^2^ Else Nutrition powder; ^3^ Largest standard error of the mean; ^4^ Repeated measures ANOVA *p*-value for the fixed effects of time, diet group, and their interactions; ^5^ *p*-value adjusted by Sidak’s multiple comparisons test representing the contrast between diet groups at each week (w) of age; CK = creatinine kinase; AST = aspartate aminotransferase; ALT = alanine transferase.

**Table 4:** Group average per week of age (w1, w2, w3) of kidney function biomarkers and electrolytes measured in the plasma of piglets fed either a dairy-based or a plant-based formula (*n* = 9/group) from postnatal day (PND) 2 until PND 21.

								*p* ^4^			*p*-Adjusted ^5^		
	Dairy-Based Formula ^1^			Plant-Based Formula ^2^			SEM ^3^	Time	Group	Group × Time	w1	w2	w3
Electrolytes	w1	w2	w3	w1	w2	w3							
Sodium (mEq/L)	135.10	137.20	136.7	129.50	135.80	137.30	1.34	<0.01	0.04	<0.01	<0.01	0.61	0.94
Potassium (mEq/L)	4.54	4.53	4.91	4.26	4.63	4.23	0.28	0.57	0.10	0.12	0.69	0.98	0.04
S:K ratio	29.83	30.48	28.66	30.59	29.86	32.50	1.57	0.90	0.19	0.08	0.95	0.97	0.04
Chloride (mEq/L)	102.30	104.90	106.70	101.80	102.00	104.00	1.08	<0.01	0.01	0.20	0.95	0.02	0.03
Kidney function													
Creatinine (mg/dL)	0.37	0.40	0.51	0.41	0.33	0.51	0.034	<0.01	0.74	0.05	0.42	0.12	1.00
BUN (mg/dL)	5.89	3.67	2.78	3.29	5.56	4.00		0.37	0.84	0.07	0.23	0.45	0.76

^1^ Similac Advance powder; ^2^ Else Nutrition powder; ^3^ Largest standard error of the mean; ^4^ Repeated measures ANOVA *p*-value for the fixed effects of time, diet group, and their interactions; ^5^ *p*-value adjusted by Sidak’s multiple comparisons test representing the contrast between diet groups at each week (w) of age; S:K = sodium:potassium ratio; BUN = plasma urea nitrogen.

**Table 5:** Concentration of minerals (group average) per week of age (w1, w2, w3) in the plasma of piglets fed either a dairy-based or a plant-based formula (*n* = 9/group) from postnatal day (PND) 2 until PND 21.

								*p* ^4^			*p*-Adjusted ^5^		
	Dairy-Based Formula ^1^			Plant-Based Formula ^2^			SEM ^3^	Time	Group	Group × Time	w1	w2	w3
Minerals	w1	w2	w3	w1	w2	w3							
Calcium (mg/dL)	10.78	10.24	9.92	11.55	10.10	9.79	0.27	<0.01	0.50	<0.01	0.02	0.93	0.94
Phosphorus (mg/dL)	5.50	7.78	8.07	5.57	7.39	7.72	0.49	<0.01	0.55	0.65	1.00	0.79	0.85
Iron (µg/dL)	254.90	371.90	507.40	555.00	492.90	249.20	190.40	0.92	0.65	0.11	0.32	0.90	0.45
Selenium (ng/mL)	171.90	161.60	153.10	191.30	167.30	162.40	16.58	0.12	0.24	0.83	0.58	0.98	0.92
Zinc (µg/mL)	1.32	1.12	1.06	1.88	0.94	0.72	0.27	<0.01	0.94	0.05	0.12	0.88	0.51
Manganese (ng/mL)	3.14	2.76	2.92	4.35	3.18	1.83	0.87	0.08	0.71	0.15	0.43	0.94	0.46
Molybdenum (ng/mL)	6.19	5.85	5.44	6.57	6.75	5.24	3.63	0.40	0.61	0.80	0.99	0.83	1.00
Cobalt (ng/mL)	0.50	0.22	0.52	1.06	0.36	0.32	0.22	0.01	0.22	0.06	0.04	0.90	0.74
Copper (ng/mL)	0.81	1.23	1.24	1.13	1.37	1.31	0.13	<0.01	0.08	0.21	0.04	0.61	0.91

^1^ Similac Advance powder; ^2^ Else Nutrition powder; ^3^ Largest standard error of the mean; ^4^ Repeated measures ANOVA *p*-value for the fixed effects of time, diet group, and their interactions; ^5^ *p*-value adjusted by Sidak’s multiple comparisons test representing the contrast between diet groups per week (w) of age.

**Table 6:** Cell plasma count variables (group average) by week of age (w1, w2, w3) of piglets fed either a dairy-based or a plant-based formula (*n* = 9/group) from postnatal day (PND) 2 until PND 21.

								*p* ^4^			*p*-Adjusted ^5^		
	Dairy-Based Formula ^1^			Plant-Based Formula ^2^			SEM ^3^	Time	Group	Group × Time	w1	w2	w3
Variable	w1	w2	w3	w1	w2	w3							
RBC (M/µL)	4.22	4.44	5.31	5.02	4.69	5.47	0.38	<0.01	0.10	0.23	0.02	0.82	0.96
Hemoglobin (g/dL)	8.63	9.15	10.65	10.01	8.80	10.46	0.63	<0.01	0.43	0.03	<0.01	0.90	1.00
Hematocrit (%)	29.06	29.44	34.50	34.44	29.21	33.66	2.08	0.01	0.22	0.02	<0.01	1.00	0.97
MCV (fL)	69.29	66.44	64.56	61.63	62.22	61.04	7.66	0.74	0.09	0.78	0.19	0.73	0.90
MCH (pg)	20.53	20.65	20.02	20.70	18.77	18.93	1.07	0.16	0.08	0.22	1.00	0.10	0.68
Plasma protein (g/dL)	5.59	5.30	5.22	7.03	5.23	5.27	0.43	<0.01	0.05	0.01	<0.01	1.00	1.00
Total WBC (k/µL)	6.15	7.00	8.61	7.24	6.93	6.30	1.27	0.63	0.49	0.11	0.57	1.00	0.22
Neutrophils (%)	37.89	42.11	34.29	48.33	45.11	33.71	5.23	0.02	0.13	0.27	0.08	0.89	1.00
Monocytes (%)	3.86	4.00	3.30	2.89	2.99	2.49	1.07	0.66	0.14	0.99	0.64	0.61	0.84
Lymphocytes (%)	56.78	52.11	61.86	47.78	50.78	63.14	5.28	0.01	0.29	0.31	0.17	0.99	0.99
Abs neutrophils (k/µL)	2.38	3.01	2.98	3.62	3.17	2.30	0.86	0.70	0.58	0.19	0.16	0.99	0.82
Abs monocytes (k/µL)	0.25	0.28	0.32	0.25	0.21	0.19	0.14	1.00	0.35	0.72	1.00	0.89	0.75
Abs lymphocytes (k/µL)	3.42	3.60	5.22	3.27	3.47	3.83	0.66	0.02	0.09	0.27	0.98	0.99	0.12

^1^ Similac Advance powder; ^2^ Else Nutrition powder; ^3^ Largest standard error of the mean; ^4^ Repeated measures ANOVA *p*-value for the fixed effects of time, diet group, and their interactions; ^5^ *p*-value adjusted by Sidak’s multiple comparisons test representing the contrast between diet groups at each week (w) of age; RBC = red blood cells; MCV = mean corpuscular volume; MCH = mean corpuscular hemoglobin; WBC = white blood cells; Abs = absolute.

## Supplementary Materials

The following are available online at https://www.mdpi.com/article/10.3390/nu14071499/s1, Table S1: Diet composition of the dairy- and plant-based milk formulas offered to the piglets from postnatal day 2 until 21.
