# Supplementary material for: Evaluation of the Safety of a Plant-Based Infant Formula Containing Almonds and Buckwheat in a Neonatal Piglet Model
Source: Nutrients. 2022 Apr 2;14(7):1499. doi: 10.3390/nu14071499 (PMC9002815; doi:10.3390/nu14071499)
Supplement: Supplementary file 1 [file nutrients-14-01499-s001.zip › nutrients-1642878-supplementary.pdf]

Supplemental Table S1. Diet composition of the dairy- and plant-based milk formulas[1] offered to the piglets from postnatal day 2 until 21.

Nutrient	Dairy-based milk formula (g/Kg)	Plant-based milk formula (g/Kg)
Test Formula, customer supplied	514.6	600
Whey Protein Isolate	200	170
L-Arginine	2	0
L-Histidine	1.9	2.1
L-Phenylalanine	1.8	5.2
Mineral Mix, w/o Ca & P (98057)	13.4	13.4
Calcium Phosphate, dibasic	27	22
Potassium Phosphate, monobasic	6.2	4
Sodium Chloride	5.7	0
Ferric Citrate	0.24	0.3
Zinc Carbonate	0.2	0.2
Sodium Selenite (0.0455% in sucrose)	0.9	0.2
Vitamin Mix, AIN-93 w/o A, D, E (120379)	10	10
Choline Chloride	0.77	0.3
Xanthan Gum	20	20
Corn Starch	16.29	1.3
Safflower Oil, linoleic	109	92
Coconut Oil	70	59

[1]Both milk formulas were mixed with safflower and coconut oils to make the complete diet. The complete diet had a caloric density of 4.89 and 5.01 Kcal/g for the dairy- and plant-based milk diets, respectively.
